# Platelet Counts and Platelet Activation Markers in Obese Subjects

**DOI:** 10.1155/2008/834153

**Published:** 2008-03-12

**Authors:** Dorit Samocha-Bonet, Dan Justo, Ori Rogowski, Nili Saar, Subchi Abu-Abeid, Galina Shenkerman, Itzhak Shapira, Shlomo Berliner, Aaron Tomer

**Affiliations:** ^1^Department of Physiology and Pharmacology, School of Medicine, Sackler Faculty of Medicine, Tel-Aviv University, 69978 Ramat-Aviv, Israel; ^2^Department of Internal Medicine D, Tel-Aviv Sourasky Medical Center, Tel-Aviv University, 6 Weitzman Street, 64239 Tel-Aviv, Israel; ^3^Obesity Center, Tel-Aviv Sourasky Medical Center, Tel-Aviv University, 6 Weizman Street, 64239 Tel-Aviv, Israel; ^4^Blood Bank and Transfusion Medicine, Soroka Medical Center, Faculty of Health Sciences, Ben-Gurion University of the Negev, 84105 Beer-Sheva, Israel

## Abstract

*Objective*. In this work we studied the correlation between platelet count, platelet activation, and systemic inflammation in overweight, obese, and morbidly obese individuals. *Methods and subjects*. A total of 6319 individuals participated in the study. Complete blood counts, high sensitivity C-reactive protein (hs-CRP) serum levels, and body mass index (BMI) were measured during routine checkups. Platelet activation markers were studied among 30 obese (BMI = 41 ± 8 kg/m^2^) and 35 nonobese (BMI = 24 ± 3 kg/m^2^) individuals. Platelet activation status was evaluated by flow cytometry using specific antibodies against the activated platelet membrane glycoprotein IIb/IIIa, p-selectin (CD-62 p), and binding of Annexin-V to platelet anionic phospholipids. *Results*. Overweight, obese, and morbidly obese females had significantly elevated platelet counts ( *P* < .0001) compared with normal-weight females. No significant elevation of platelet counts was observed in the male subgroups. A significant age adjusted correlation between BMI and platelet counts ( *P* < .0001) was found among females. This correlation was attenuated (*P* = .001) after adjustment for hs-CRP concentrations. The flow cytometry analysis of platelets showed no significant differences in activation marker expression between nonobese and obese individuals. *Discussion*. Obesity may be associated with elevated platelet counts in females with chronic inflammation. Obesity is not associated with increased platelet activation.

## 1. INTRODUCTION

Obesity, a major risk factor for cardiovascular disease, is associated with an accelerated atherothrombotic process, resulting in increased morbidity and mortality [1]. Cytokines, such as Interleukin-6 (IL-6), originating from adipose tissue, have a fundamental role in the pathogenesis of atherothrombosis [2]. IL-6 acts synergistically with other interleukins, growth factors, and thrombopoietin in megakaryocytopoiesis; in-vivo administration of IL-6 to both monkeys and humans reportedly increased circulating platelet counts [3]. Higher platelet counts are associated with adverse clinical outcome in patients with ST-elevation myocardial infarction [4]. The association between increased platelet counts and platelet activation is unclear; in patients with carotid stenosis, increased platelet counts, and leukocyte-platelet complex formation, that is, platelet activation, was associated with the risk of stroke [5]; platelet count and platelet activation are associated in patients with chronic inflammation, such as patients with essential thrombocytosis [6], and patients with inflammatory bowel diseases [7]. Obesity is a chronic inflammation state [2], yet the association between platelet count and platelet activation has never been studied in obese subjects, to the best of our knowledge.

There is an ongoing debate on whether obesity is accompanied by platelet activation. The findings that the leptin receptor is expressed in platelets [8] and that leptin potentiates platelet aggregation by agonists [8, 9] shed light on a possible direct link between obesity and thrombotic complication. Davi et al. demonstrated platelet activation in obese individuals by measuring urine excretion of a thromboxane B_2_ (TxB_2_) metabolite (11-dehydrothromboxane B_2_, 11-dehydro TxB_2_) [10]. In contrast, other studies found no evidence of increased platelet activation in obese individuals [11], in obese and overweight women [12], in overweight prediabetics [13], and further reported a negative correlation between plasma *β*-thromboglobulin (BTG), a marker of platelet release, and body weight [14]. In the present study, we studied platelet count and platelet activation in obese subjects.

## 2. MATERIALS AND METHODS

### 2.1. Patients

Participants were all part of the Tel-Aviv Medical Center Inflammation Survey (TAMCIS). The TAMCIS is a cross-sectional study performed on a group of apparently healthy individuals, consisting of employees of the Tel-Aviv Sourasky Medical Center (TASMC) and the Tel-Aviv Municipality. The study was conducted between September 2002 and July 2006. All patients gave their informed consent to participate in the survey in writing, according to the local ethics committee instructions. Recruitment for the study was performed through announcements in the monthly salary slips of the Tel-Aviv Medical Center personnel. Platelet count was studied initially in 9115 subjects. Excluded were individuals with an underlying chronic inflammatory disease (*n* = 1677), such as arthritis, inflammatory bowel disease, individuals with an acute or recent inflammatory condition, and individuals with a history of myocardial infarction or surgery during the past six months. Also excluded were individuals treated with antiplatelets, anticoagulants, hormone replacement therapy, oral contraception, systemic steroids, immunosuppressive agents, and nonsteroidal anti-inflammatory drugs (*n* = 1119). Medical conditions were self-reported, but were confirmed by a physician who took history and examined each individual. Platelet activation was studied in 65 randomly assigned patients: 30 obese subjects from the obesity clinic and 35 nonobese subjects from the general study. In this group, subjects taking antiplatelets, hormone replacement therapy, and oral contraception were not excluded.

### 2.2. Cardiovascular risk factors

The diagnosis of diabetes mellitus was consistent with the guidelines of the American Diabetic Association. Diabetic individuals were those with fasting plasma glucose level of 126 mg/dL or higher, or those who were taking hypoglycemic agents [15]. The diagnosis of essential hypertension was consistent with the seventh report of the Joint National Committee on prevention, detection, evaluation, and treatment of high blood pressure (JNC 7). Hypertensive individuals were those with systolic blood pressure (BP) of 140 mmHg or higher and/or a diastolic BP of 90 mmHg or higher repeatedly, or individuals who were taking antihypertensive agents [16].

### 2.3. Metabolic syndrome definition

Metabolic syndrome was defined as having at least three of the following: men with high-density lipoprotein (HDL) cholesterol ≤ 40 mg/dL, women with HDL cholesterol ≤ 50 mg/dL, triglyceride ≥ 150 mg/dL for both genders, blood pressure ≥ 130/85 mmHg for both genders, fasting plasma glucose (FBG) ≥ 110 mg/dL for both genders, and waist circumference ≥ 102 cm for men and ≥ 88 cm for women [15]. Fasting glucose levels and lipid profiles were measured by routine biochemical determinations. Blood samples for plasma glucose levels and lipid profile were drawn after an overnight fast from all individuals.

### 2.4. Platelet counts and inflammatory markers

Complete blood counts were performed using the Coulter STKS (Beckman Coulter, Nyon, Switzerland) automatic cell analyzer. High-sensitivity C-reactive protein (hs-CRP) was measured using the Boering BN II nephelometer (DADE Boering, Marburg, Germany) according to Rifai et al. [17]. Blood samples for complete blood counts and systemic inflammation markers were drawn after an overnight fast from all individuals.

### 2.5. Platelet activation markers

Platelet activation was studied as previously described [18, 19]. Briefly, blood was collected in citrate-containing syringes (1 : 10 volume of 3.8% citrate) and processed immediately to prevent possible in-vitro activation of platelets. Platelet-rich plasma was prepared immediately by standard slow centrifugation ( 150× g for 12 minutes) and used for flow cytometry analysis of platelet activation markers. A 5 *μ*L platelet suspension ( 250 × 10^9^ platelets/L) was immediately incubated with Ca^++^- and Mg^++^-free phosphate-buffered saline (PBS) and monoclonal antibodies (MoAb) in saturating concentrations, in a total volume of 50 *μ*L. An HBSS-HEPES buffer containing 2.5 mM CaCl_2_ was used for the incubation with Annexin-V. Following 30 minutes incubation at 4°C, the samples were diluted 1 : 10 with the same buffer and analyzed by a FACSCalibur flow cytometer (Becton Dickinson Biosciences, Calif, USA). The samples were not lysed or fixed and were analyzed immediately. MoAbs included phycoerythrin (PE)-labeled CD41 against resting and activated glycoprotein IIb/IIIa (Immunotech, Marseille, France), fluorescein-isothiocyanate (FITC)-labeled PAC-1 against the activated conformation of glycoprotein IIb/IIIa (Becton Dickinson Biosciences, CA), FITC-labeled P-selectin (CD62p), an *α*-granule membrane glycoprotein expressed on the platelet surface during secretion (Immunotech, Marseille, France), and FITC-labeled annexin-V, which is known to react with platelet anionic-phospholipids, such as phosphatydilserine (PS) exposed on platelets following activation (R&D Systems, Minneapolis, MN). Isotype-matched MoAbs (Immunotech, France; DAKO, DK) were used as negative controls, and platelets treated in-vitro with adenosine diphosphate (ADP) and Ca^++^-Ionophore A23817 (final concentrations 20 *μ*mol/L and 5 *μ*mol/L, resp.) were used as positive controls. Platelets were identified by light-scatter properties and CD41 expression and analyzed for binding of the specific Ab. All measurements were performed on list mode using logarithmic scales and 10,000 platelets were analyzed in each sample using the Cellquest Pro software (Becton Dickinson). The results are expressed as mean fluorescence intensity (MFI) units for the studied markers of activation.

### 2.6. Statistical analysis

For platelet count analysis, subjects were divided into four groups based on their BMI: normal weight (BMI < 25), overweight (25 < BMI < 29.9), obese (30 < BMI < 39.9), and morbidly obese (BMI ≥ 40). Data was analyzed separately for males and females due to gender differences in baseline inflammatory profiles and platelet counts [20]. Differences in prevalence of cardiovascular risk factors and cardiovascular disease between the various BMI subgroups were analyzed using the chi-square test for discrete variables, and analysis of variance (ANOVA) with the general linear model for continuous variables. Since hs-CRP had a non-normal distribution to begin with, a logarithmic transformation of hs-CRP was used for all statistical procedures. Differences between the BMI subgroups in terms of platelet counts, hemoglobin levels, and hs-CRP levels were analyzed using ANOVA. Platelet counts, hemoglobin levels and hs-CRP levels for overweight, obese, and morbidly obese subgroups were compared with those of the normal-weight subgroup using the general linear model with post hoc multiple comparisons by the method of Scheffe. The student's *t* test was used to evaluate differences in platelet counts between subjects with or without the metabolic syndrome. ANOVA was used to evaluate the association between BMI and platelet counts after age- and hs-CRP-adjustments. For platelet activation analysis, 65 subjects were divided into two groups based on their BMI: nonobese (BMI < 30) and obese (BMI ≥ 30). The student's *t* test and Mann-Whitney test were used to evaluate differences in the studied markers between the two groups. BMI and waist-to-hip ratio were normally distributed in both groups. The SPSS statistical package was used (SSPS Inc., Chicago, IL, USA).

## 3. RESULTS

### 3.1. Platelet counts and BMI status

Platelet counts were studied among 6319 individuals, 4352 males and 1967 females. The mean age of the cohort was 44.6 ± 10.4 years. Overall, 1234 (19.5%) subjects had hypertension, 246 (3.9) subjects had diabetes mellitus, 1923 (30.4%) had dyslipidemia, 85 (1.3%) had history of ischemic heart disease, and 9 (0.1%) had history of ceberovascular accident. Overall, 2463 (39.0%) were normal weight, 2749 (43.5%) were overweight, 1058 (17%) were obese, and 49 (0.8%) were morbidly obese. The prevalence of hypertension, diabetes mellitus, and history of myocardial infarction significantly increased with BMI category (Table 1).

Platelet counts increased with BMI in both genders. However, only among females, the platelet counts were significantly elevated in the overweight (*P* = .015), obese (*P* < .0001), and morbidly obese (*P* < .0001) subgroups compared with the normal-weight subgroup after adjustment for age, diabetes mellitus, and hypertension. Using ANCOVA, platelet counts were still associated with BMI among females after adjustment for age and hs-CRP (*P* = .034). Platelet counts were elevated, though not statistically significant, in the overweight, obese, and morbidly obese male subgroups compared with the normal-weight subgroup. The association between obesity and inflammation was apparent by an increment in hs-CRP concentrations with BMI categories in both males and females (Table 2). Mean platelet counts were significantly higher in obese females with the metabolic syndrome compared with obese females without the metabolic syndrome (*P* = .032). In the overweight and morbidly obese female subgroups, the platelet counts tended to be higher in women with the metabolic syndrome, however, this tendency did not reach statistic significance (Table 3). Finally, there was a significant age-adjusted correlation between BMI and platelet counts (*P* < .0001) in females. This correlation was attenuated but remained significant (*P* = .001) after adjustment for hs-CRP concentrations.

### 3.2. Platelet activation and BMI status

Platelet activation was studied in 65 individuals, 49 females, and 16 males. They were divided according to their BMI levels into nonobese (BMI < 30, *n* = 35) and obese (BMI ≥ 30, *n* = 30) subjects. Their clinical characteristics and laboratory findings are summarized in Table 4. Nine of the obese subjects were on antihypertensive drugs and 3 were on low-dose aspirin therapy. In the nonobese subgroup, 7 subjects were on estrogen treatment (oral contraceptives or hormone replacement therapy), and 4 were on low-dose aspirin therapy. The obese group differed from the nonobese group in fat distribution, as evidenced by larger waist circumferences and larger waist-to-hip ratios. Significant differences between obese and nonobese individuals were also found in the metabolic markers FBG, HDL-cholesterol and serum triglyceride concentrations. Table 4 displays the platelet activation status as measured by the expression of the MoAb studied. No significant differences were found in platelet activation status between the groups. Additionally, no statistically significant correlation was found between BMI or waist-to-hip ratios and indices of platelet activation (Table 5).

## 4. DISCUSSION

It has been well established that obesity is associated with low-grade subclinical and smoldering inflammation [2, 21, 22]. Both BMI and body fat mass have been shown to correlate with total leukocyte counts [23]. The Atherosclerosis Risk in Community (ARIC) study demonstrated a positive correlation between leukocyte and platelet counts [24], but no significant correlation between obesity and platelet counts. Moreover, it has not yet been established whether elevated platelet counts in obese individuals are associated with platelet activation. The answer to this question is especially relevant when primary prevention by antiplatelet therapy is considered for individuals at risk.

The results of the present study demonstrate an association between obesity and platelet counts in females probably due to higher body fat mass. Yudkin et al. [21] have demonstrated an association between obesity and IL-6 levels. A large proportion of IL-6 in the circulation originates from the adipose tissue that in turn may contribute to atherogenesis and thrombosis, by promoting inflammation. One of the proposed mechanisms for IL-6 contribution to atherogenesis and thrombosis is its effect on platelets, fibrinogen concentrations, and coagulation [2]. IL-6, in addition to other interleukins, plays a crucial role in the proliferation of megakaryocyte progenitors and acts synergistically with thrombopoietin and stem cell factor in stimulating megakaryocytopoiesis [3, 25, 26]. Thus it is conceivable that the elevated platelet counts found in obese females in the present study are secondary to the presence of a chronic inflammation as evident by elevated hs-CRP levels. In fact, the correlation between BMI and platelet counts found in females was attenuated once adjusted for hs-CRP levels.

The ambiguity found in the literature regarding the role of platelet activation in obese individuals stems from employment of different markers to evaluate platelet activation in previous studies. Tangorra et al. [27] and Meade et al. [28] used aggregating agents to test platelet susceptibility to aggregation. Both groups showed no correlation between obesity and the susceptibility of platelets to aggregating agents. Davi et al. and Licata et al. used 11-dehydro-TxB_2_ excreted in urine to evaluate platelet activation. In an earlier (relatively small) study, they found no differences in 11-dehydro-TxB_2_ excretion between obese and nonobese individuals [11], yet later [10], the same group reported increased levels of excreted 11-dehydro-TxB_2_ in obese compared to nonobese women associated with android obesity. Interestingly, both *β*-thromboglobulin and the metabolite 11-dehydro-TxB_2_ correlate positively with platelet counts [29, 30], and increased concentrations of these, found in obese individuals, may simply reflect their, already discussed, elevated counts. Dogru et al. and De Pergola et al. used soluble P-selectin (sP-selectin) plasma levels as a marker of platelet activation and found either similar level in overweight prediabetic and healthy individuals [13] or increased sP-selectin in overweight and obese compared to normal-weight individuals [12]. The latter finding failed to maintain significant associations in a multiple regression that accounted for various anthropometric, metabolic and inflammation markers [12]. In the present study, we used a panel of sensitive flow cytometry determinants not previously reported in apparently healthy obese and nonobese individuals. Flow cytometry is particularly well suited for discriminating between various early activation events. Flow cytometry is highly sensitive in its ability to demonstrate expression of active membrane epitopes preceding early activation-induced platelet morphological changes. This method is also capable of detecting activated platelets in the circulation despite their rapid clearance [18, 19, 31]. The present study demonstrates no evidence of increased platelet activation in obese subjects despite the fact that obese and morbidly obese subjects exhibited chronic inflammation. Our results are consistent with those of Dogru et al. and De Pergola et al. [12, 13] and with the study published by Marquardt et al. [32] that found no correlation between inflammation markers including leukocyte count, CRP and fibrinogen levels, and platelet membrane p-selectin (CD62p) expression in patients after an ischemic stroke.

## Figures and Tables

**Table 1:** Clinical characteristics of subjects for platelet count analysis stratified by BMI.

BMI	<25	25–29.9	30–39.9	>40	*P*-value
*n*	2460	2749	1057	49	—
Age (mean ± SD)	41.8 ± 10.6 yrs	45.9 ± 10.1 yrs	47.7 ± 9.0 yrs	45.6 ± 7.6 yrs	<.0001
Hypertension	246 (10.0%)	596 (21.7%)	370 (35%)	22 (44.9%)	<.0001
Diabetes mellitus	38 (1.5%)	91 (3.3%)	106 (10.0%)	11 (22.4%)	<.0001
Dyslipidemia	451 (18.3%)	911 (33.1%)	451 (51.1%)	20 (40.8%)	<.0001
Ischemic heart disease	30 (1.2%)	35 (1.3%)	19 (1.8%)	1 (2.0%)	.53
Past CVA	4 (0.2%)	1 (0.0%)	3 (0.3%)	1 (0.02%)	.001

BMI = body mass index; MI = myocardial infarction; CVA = cerebrovascular accident.

**Table 2:** Age, hypertension, and diabetes mellitus adjusted estimated marginal mean (standard error of the mean) of platelets count, hemoglobin concentration, and CRP stratified by BMI.

Body mass index	<25	25–29.9	30–39.9	>40	*P*-value
*Females*					
* *N	1015	585	341	26	—
* *Platelet count ( × 10^9^/L)	260 (4)	270 (4)*	281 (4)*	307 (11)*	<.0001
* *Hemoglobin (g/L)	13.2 (0.08)	13.2 (0.08)	13.2 (0.08)	13.2 (0.21)	.893
* *hs-CRP	1.2 (1.1)	2.5 (1.1)*	5.2 (1.1)*	9.3 (1.2)*	<.0001
*Males*					
* *N	1448	2164	717	23	—
* *Platelet count (× 10 ^9^/L)	241 (3)	242 (2)	246 (3)	260 (11)	.11
* *Hemoglobin (g/L)	14.8 (0.04)	15.0 (0.04)*	15.0 (0.05)*	14.8 (0.20)	<.0001
* *hs-CRP	1.1 (1.0)	1.7 (1.0)*	2.6 (1.0)*	6.2 (1.2)*	<.0001

*P < .05 compared with the BMI <25 group.

**Table 3:** Platelet counts in overweight, obese, and morbidly obese females with and without the metabolic syndrome.

	Metabolic syndrome	No metabolic syndrome	*P*-value
BMI 25–29.9 (*n* = 585)	270 ± 67 (*n* = 73)	260 ± 56 (*n* = 512)	.23
BMI 30–39.9 (*n* = 341)	283 ± 62 (*n* = 119)	268 ± 60 (*n* = 222)	.032
BMI > 40 (*n* = 26)	314 ± 53 (*n* = 14)	292 ± 52 (*n* = 12)	.29

BMI = body mass index.

**Table 4:** Clinical data, laboratory findings, and platelet activation markers for subjects in the platelet activation analysis.

	Nonobese	Obese	*P*-value
*n*	35	30	—
Age (years)	43 ± 12	41 ± 13	.2
Body mass index (kg/m^2^)	24 ± 2.7	41 ± 7.7	<.0001
Waist circumference (cm)	83 ± 12	120 ± 14	<.0001
Hip circumference (cm)	96 ± 6	131 ± 19	<.0001
Waist/hip ratio	0.86 ± 0.13	0.93 ± 0.15	.06
Blood glucose (mg/dl)	82 ± 8	105 ± 26	.003
HDL cholesterol (mg/dl)	62 ±17	46 ± 12	<.0001
Triglyceride levels (mg/dl)	119 ± 63	170 ± 87	.01
Total cholesterol (mg/dl)	232 ± 49	216 ± 47	.2
LDL cholesterol (mg/dl)	148 ± 47	136 ± 41	.3
Platelet count ( × 10^9^/L)	259 ± 55	263 ± 55	.7
*Platelet activation markers* (MFI, range given in parentheses)			
PAC-1 binding	8.4 ± 5.9 (1.8–24.6)	6.5 ± 4.7 (1.7–22.7)	.09
P-selectin (CD-62p)	2.1 ± 0.8 (1.3–4.4)	2.2 ± 0.6 (1.5–3.6)	.3
Annexin-V binding	4.4 ± 0.6 (2.1–12.2)	4.7 ± 1.5 (2.4–8.0)	.15

HDL = high-density lipoprotein; LDL = low-density lipoprotein; MFI = mean fluorescence intensity.

**Table 5:** Correlations between platelet activation markers, body mass index, and waist-to-hip ratios for subjects in the platelet activation analysis.

Platelet activation markers	BMI (kg/m^2^)	Waist-to-hip ratio
*n*	65	65
PAC-1 binding	*r* = −0.2	*r* = 0.004
	*P* = .1	*P* = .9
CD62p expression	*r* = −0.05	*r* = −0.12
	*P* = .7	*P* = .4
Annexin-V binding	*r* = 0.06	*r* = −0.15
	*P* = .6	*P* = .3

BMI = body mass index; MFI = mean fluorescence intensity.
